# A78 VALIDITY EVIDENCE FOR OBSERVATIONAL EUS COMPETENCY ASSESSMENT: A SYSTEMATIC REVIEW

**DOI:** 10.1093/jcag/gwad061.078

**Published:** 2024-02-14

**Authors:** A Ceccacci, H Hothi, R Khan, N Gimpaya, B Chan, N Forbes, P D James, J Mosko, E Yeung, C M Walsh, S C Grover

**Affiliations:** University of Toronto, Toronto, ON, Canada; University of Toronto, Toronto, ON, Canada; University of Toronto, Toronto, ON, Canada; Unity Health Toronto, Toronto, ON, Canada; Scarborough Health Network, Scarborough, ON, Canada; University of Calgary, Calgary, AB, Canada; University Health Network, Toronto, ON, Canada; Unity Health Toronto, Toronto, ON, Canada; Scarborough Health Network, Scarborough, ON, Canada; The Hospital for Sick Children, Toronto, ON, Canada; Unity Health Toronto, Toronto, ON, Canada

## Abstract

**Background:**

Endoscopic ultrasound (EUS) encompasses a range of diagnostic and therapeutic procedures that require technical, cognitive, and non-technical skills. The implementation of competency-based frameworks in endoscopic education has emphasized trainee assessment based on predefined milestones, rather than procedure volume. Observational assessment tools with strong validity evidence are needed to achieve this goal.

**Aims:**

To systematically identify and evaluate observational competency assessment tools employed in EUS using an established validity framework. The secondary aim is to evaluate the educational utility of assessment tools.

**Methods:**

We searched three databases (MEDLINE, EMBASE, and Evidence-Based Medicine Reviews) and the grey literature from inception to May 2023. Messick’s unified framework was used to evaluate validity evidence based on content, response process, internal structure, relations to other variables, and consequences. Each domain was scored from 0 to 3 with a maximum score of 15 points. Educational utility was evaluated using the Accreditation Council for Graduate Medical Education Standards considering ease of use, ease of interpretation, resources required, and educational impact. Study quality was assessed using the Medical Education Research Quality Instrument.

**Results:**

Our search identified 2081 records. We screened 44 full texts and included 5 observational EUS assessment tools from 10 studies. All 5 tools are formative assessments, with 4 employed in clinical settings and one in a simulated setting. All tools use Likert rating scales and are rater-based, with 2 having additional self-assessment components. Validity evidence scores ranged from 3 to 13, with the EUS Assessment Tool (EUSAT), Global Assessment of Performance and Skills in EUS (GAPS-EUS), and The EUS and ERCP Skills Assessment Tool (TEESAT) scoring highest, with 10, 11, and 13 points, respectively. Overall educational utility was high across studies given ease of tool use. The TEESAT had the strongest educational impact considering its influence on credentialing and competence thresholds. Study quality was high overall, with scores ranging from 9.5 to 12 (maximum 13.5 points). Inter-rater agreement for validity evidence and educational utility scoring was substantial (k=0.73, raw agreement 80%) and almost perfect (k=0.92, raw agreement 96%), respectively.

**Conclusions:**

The EUSAT, GAPS-EUS, and TEESAT demonstrate the strongest validity evidence for observational competency assessment of EUS and are easy to implement in educational settings. Future work should investigate barriers to implementation and evaluate utility of these tools for summative assessment.

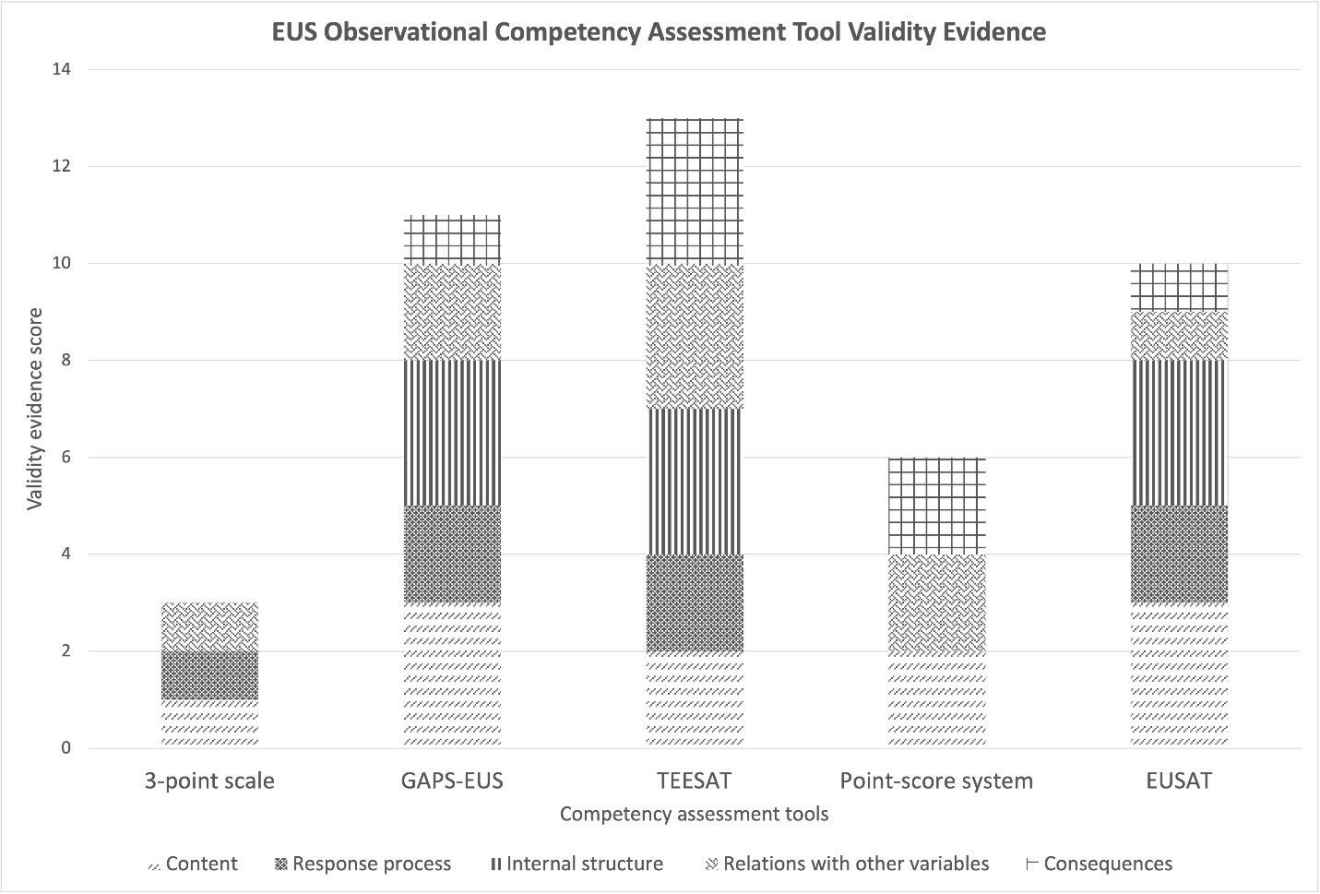

EUS Observational Competency Assessment Tool Validity Evidence Scores

**Funding Agencies:**

None

